# Validation of the effectiveness of a digital integrated healthcare platform utilizing an AI-based dietary management solution and a real-time continuous glucose monitoring system for diabetes management: a randomized controlled trial

**DOI:** 10.1186/s12911-020-01179-x

**Published:** 2020-07-10

**Authors:** Sung Woon Park, Gyuri Kim, You-Cheol Hwang, Woo Je Lee, Hyunjin Park, Jae Hyeon Kim

**Affiliations:** 1 Division of Endocrinology and Metabolism, Department of Internal Medicine, CHA Gangnam Medical Center, CHA University School of Medicine, Seoul, South Korea; 2 Division of Endocrinology and Metabolism, Department of Internal Medicine, Samsung Medical Center, Sungkyunkwan University School of Medicine, Seoul, South Korea; 3 Division of Endocrinology and Metabolism, Department of Internal Medicine, Kyung Hee University Hospital at Gangdong, Kyung Hee University School of Medicine, Seoul, South Korea; 4 Division of Endocrinology and Metabolism, Department of Internal Medicine, Asan Medical Center, University of Ulsan College of Medicine, Seoul, South Korea; 5 Amazing Food Solution, Inc., Seoul, South Korea

**Keywords:** Digital healthcare, Diabetes Management, CGMS, Dietary Management, Verification of clinical trial effects

## Abstract

**Background:**

Despite the numerous healthcare smartphone applications for self-management of diabetes, patients often fail to use these applications consistently due to various limitations, including difficulty in inputting dietary information by text search and inconvenient and non-persistent self-glucose measurement by home glucometer. We plan to apply a digital integrated healthcare platform using an artificial intelligence (AI)-based dietary management solution and a continuous glucose monitoring system (CGMS) to overcome those limitations. Furthermore, medical staff will be performing monitoring and intervention to encourage continuous use of the program. The aim of this trial is to examine the efficacy of the program in patients with type 2 diabetes mellitus (T2DM) who have HbA1c 53–69 mmol/mol (7.0–8.5%) and body mass index (BMI) ≥ 23 mg/m^2^.

**Methods:**

This is a 48-week, open-label, randomized, multicenter trial consisting of patients with type 2 diabetes. The patients will be randomly assigned to three groups: control group A will receive routine diabetes care; experimental group B will use the digital integrated healthcare platform by themselves without feedback; and experimental group C will use the digital integrated healthcare platform with continuous glucose monitoring and feedback from medical staff. There are five follow-up measures: baseline and post-intervention at weeks 12, 24, 36, and 48. The primary end point is change in HbA1c from baseline to six months after the intervention.

**Discussion:**

This trial will verify the effectiveness of a digital integrated healthcare platform with an AI-driven dietary solution and a real-time CGMS in patients with T2DM.

**Trial registration:**

Clinicaltrials.gov NCT 04161170, registered on 08 November 2019.

https://clinicaltrials.gov/ct2/show/NCT04161170?term=NCT04161170&draw=2&rank=1

## Background

Diabetes mellitus is one of the most common chronic diseases and incurs extensive social and economic costs. According to the International Diabetes Federation (IDF), there were approximately 463 million people (20–79 years) with diabetes globally in 2019, and the number is expected to rise to 700 million by 2045 [1]. It is widely known that not only medications, but also lifestyle managements such as diet, exercise, and weight control, are essential in the treatment of diabetes [2–4]. Recently, advances in mobile technology have led to a large number of smartphone applications (apps) that aim to facilitate self-management of diabetes mellitus (DM) [5–8].

Although numerous healthcare apps have been developed for patients with diabetes, few of them are consistently used due to limitations. First, it is inconvenient to input multiple dietary data points. Dietary control is important in diabetes management, so feedback based on the dietary record is required. However, patients often struggle to record their dietary information reliably because it is difficult to search and enter every food item eaten into the smartphone healthcare apps. In addition, it is hard to convert the various entered foods into actual dietary data such as nutritional composition and calories. Moreover, because values of blood glucose measured by a home glucometer have to be manually entered into the apps, there are multiple opportunities for typographical errors in the glucose records. Recently, Bluetooth technology has allowed for values of blood glucose by a glucometer to be automatically recorded in any linked smartphone apps. However, due to the discomfort of the finger-prick blood test, patients do not often measure their blood glucose level. Also, most diabetes self-management apps require users to assess their body weight, blood pressure, and amount of exercise using a scale, sphygmomanometer, and pedometer, respectively, and then manually input those values. Although some healthcare apps can now be linked to devices via Bluetooth so that health information is automatically recorded into the apps, various biometric data such as blood glucose level, body weight, blood pressure, and amount of exercise are not integrated and must be provided separately for management of diabetes. In addition, most patients are inconsistent in use of these apps on their own without feedback from medical staff.

Therefore, in this study, we plan to verify the clinical effect of the digital integrated healthcare platform, which is designed to overcome these limitations using clinical intervention. First, using an artificial intelligence (AI)-driven diet management program called “FoodLens,” the only input is a single photograph of the meal prior to eating; the program then identifies all the food contents and determines the individual amounts of each in the photograph. There is no need for text search, making it considerably more convenient for users. Considering the low rate of self-assessed blood glucose level, continuous blood glucose monitoring (CGM) for 1 week every 3 months will be performed to provide blood glucose level without a finger prick. A Bluetooth-enabled glucometer, scale with bioelectrical impedance analysis, sphygmomanometer, and watch-type pedometer are each paired with the integrated platform to automatically input biometric data such as blood glucose level, body weight, body fat mass, blood pressure, and amount of exercise into the integrated platform, “Auto-check.” All the collected health information will be monitored by medical staff, who will regularly provide personal education and feedback regarding weight control, dietary plan, and exercise to the subjects.

The aim of this study is to investigate the efficacy of a digital, integrated healthcare platform using an AI-based dietary management solution and a continuous glucose monitoring system in patients with type 2 diabetes mellitus (T2DM).

## Methods

### Hypotheses

Our primary hypothesis is that the integrated healthcare platform user group with or without monitoring and feedback by medical staff will show more improved hemoglobin A1c after 6 months of intervention compared to the routine care group. Our secondary hypothesis is that the healthcare platform user group will show greater improvement in HbA1c after 12 months of intervention, improved glycometabolic parameters (Diabetes Treatment Satisfaction Questionnaire [DTSQ] score, body weight, body fat mass, amount of exercise, dietary information, lipid profile, and CGM metrics), and fewer hypoglycemic events or complications after 6 or 12 months of intervention relative to the routine care group.

### Study design

**Fig. 1:**
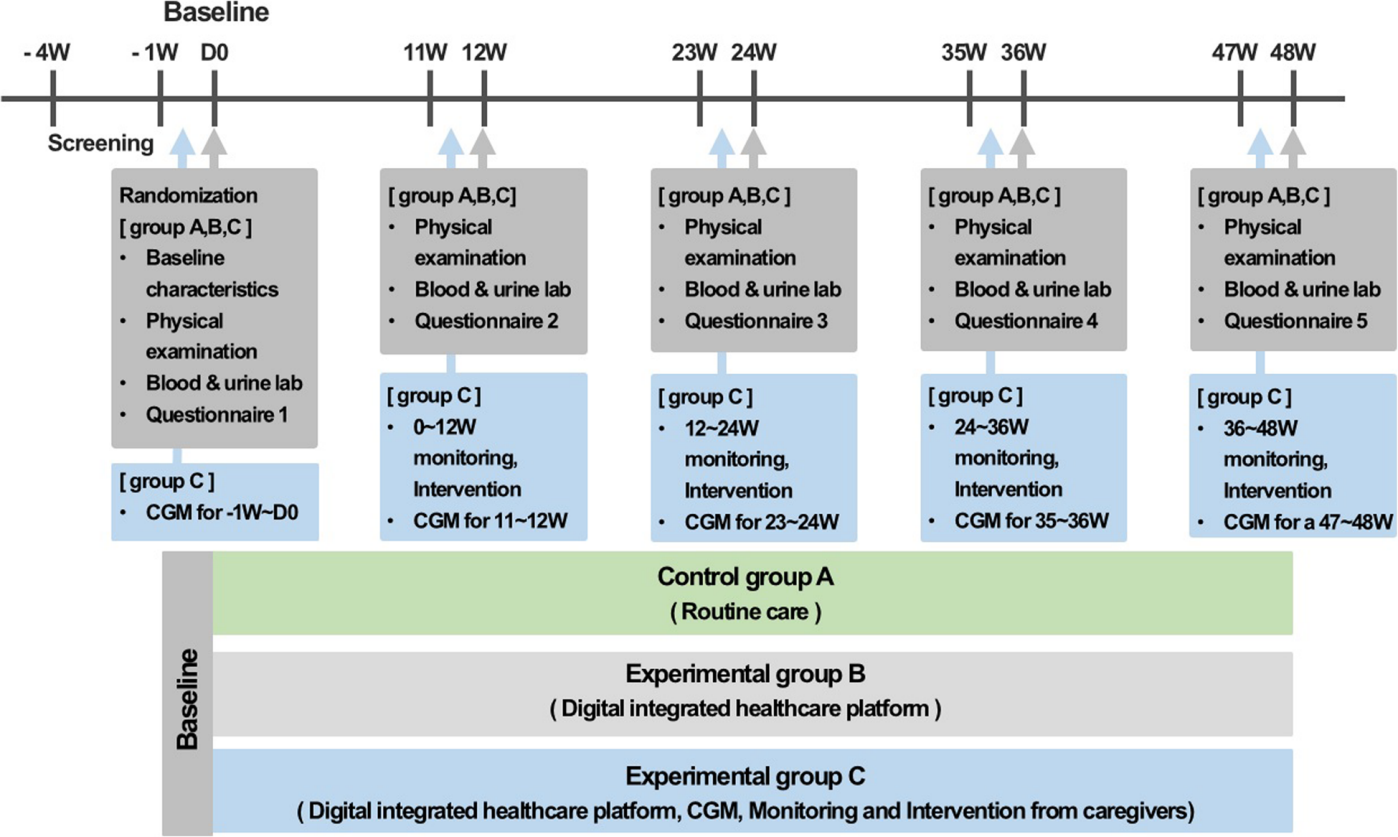
Study design. The study group includes 3 groups: Control group A (no intervention and conventional diabetes management), Experimental group B (applying the digital integrated healthcare platform by themselves, no monitoring and feedback from medical staff), and Experimental group C (applying the digital integrated healthcare platform with monitoring and feedback from medical staff and applying a CGMS). This parallel study will be conducted for 48 weeks

This is a 48-week, open-label, randomized, multicenter clinical trial conducted in three different university-affiliated hospitals in Seoul, Republic of Korea: Samsung Medical Center, Asan Medical Center, and Kyung Hee University Hospital. Subjects with T2DM will be randomly assigned to three groups in a 1:1:1 ratio. The control group “A” will receive routine diabetes care, with hospital visits every 3 months. Subjects in experimental group “B” will be introduced to the digital integrated healthcare platform to use at home without monitoring or assistance from medical staff. Subjects in experimental group “C” will be introduced to and educated on the healthcare platform for use and application at the hospital. Group C, unlike other groups, will also apply a CGMS for 1 week every 3 months. The medical staff will monitor the integrated healthcare data of group C subjects, including body weight, blood glucose, amount of exercise, and diet information, and will provide personal educational feedback via text message once a week. All subjects will visit a hospital at baseline and every 12 weeks thereafter for a total of 48 weeks (i.e., at week 12, 24, 36, and 48) and undergo a physical examination, blood and urine laboratory tests, and a Diabetes Treatment Satisfaction Questionnaire (DTSQ) at every visit. Figure 1 shows an overview of the study.

### Digital integrated healthcare platform

The mobile healthcare platform “AutoChek Care” (Aprilis Co., Ltd.) is connected via Bluetooth to a glucometer, a scale with bioelectrical impedance analysis, a sphygmomanometer, and a watch-type pedometer. The collected health information from each device, including blood glucose level, body weight, body fat mass, blood pressure, number of steps, burned calories, and total walking distance, are automatically transmitted to the app. Because all the health information obtained by several devices as required for diabetes management is linked to one smartphone application, patients and clinicians can easily access the information and analyze the data.

### AI-based dietary management

The food recognition solution “FoodLens” (DoingLab Co., Ltd.) is an AI-based program that is able to recognize dozens of foods in a single photograph. Because it provides recognition of multiple food contents in a single photograph, patients do not need to take pictures of each food item individually. The program, which is based on deep learning technology of object detection and convolution neural networks, achieved a high recognition rate of 86.6% in food classification and the recognition rate was approved by “Korea Information Security Technology.” Data from this AI-based dietary program also are incorporated into the digital integrated healthcare platform.

### Continuous glucose monitoring system

Subjects in experimental group C will apply a CGMS (Dexcom G5^Ⓡ^) for 1 week every 3 months. The glucose concentration in the interstitial fluid is measured by a sensor attached to the skin and sent to the smartphone through a wireless transmitter using Bluetooth 4.0. The installed application, “Dexcom G5 Mobile App,” displays the measured values of glucose in the interstitial fluid on the screen every 5 min, provides changing patterns of glucose values, and alerts if the glucose level falls below or rises above the set values.

### Active monitoring and feedback by medical staff

For experimental group C, a medical staff member (a clinical dietitian from Amazing Food Solution Inc., Seoul, Korea) will introduce the platform to subjects, help them to configure the devices and app, and apply the platform to the patients at the hospital. They will motivate patients to maintain their self-care through ongoing personal monitoring and intervention. Educational intervention by text message regarding glucose level, weight control, dietary plan, and exercise plan will be conducted once a week.

### Participants

#### Eligibility criteria

The inclusion criteria are as follows: 1) aged ≥19 and < 70 years; 2) diagnosis of T2DM; 3) no prescription for any hypoglycemic agent within the previous 4 weeks or taking a consistent dose of one or more oral hypoglycemic agents for more than 12 weeks; 4) most recent measurements of hemoglobin A1c ≥ 53 and ≤ 69 mmol/mol within the previous 3 months; 5) body mass index (BMI) ≥ 23.0 kg/m^2^; 6) consent to utilize the digital integrated healthcare platform for diabetes care and research; and 7) voluntary signature of the informed consent form after being informed of the clinical trial. Exclusion criteria are as follows: 1) other types of DM including type 1 DM and gestational DM; 2) use of insulin or GLP-1 agonist injection; 3) history of uncontrolled chronic liver diseases such as hemochromatosis, hepatocellular carcinoma, autoimmune liver disease, liver cirrhosis, viral hepatitis (including hepatitis A, B, and C), or Wilson’s disease; 4) history of acute kidney injury (≥ 1.5 times increased serum creatinine); 5) history of psychological disorder such as schizophrenia, depression, or bipolar disorder; 6) use of anti-obesity medication; 7) history of alcoholism or drug addiction within the 3 months prior to screening; 8) use of drugs that affect glucose metabolism, such as corticosteroids and immunosuppressive drugs, within the 3 months prior to screening; 9) pregnancy, lactation, or planning for pregnancy; and 10) other problems that investigators consider to be inappropriate for participation in the trial.

### Recruitment

Subjects will be patients diagnosed with T2DM at one of three tertiary hospitals in Seoul, Republic of Korea: Samsung Medical Center, Asan Medical Center, and Kyung Hee University Hospital. Patients who voluntarily agree to participate in the clinical trial and who meet the inclusion criteria and none of the exclusion criteria will be enrolled. All participants will receive written information about the clinical trial and must provide written informed consent.

### Outcome variables

The primary outcome will be change in HbA1c from baseline to 6 months after intervention.

The main secondary outcome will be levels of HbA1c and fasting blood glucose at weeks 12, 24, 36, and 48. Other secondary outcomes include lipid profile (i.e., total cholesterol, high-density lipoprotein [HDL] cholesterol, triglycerides, and low-density lipoprotein [LDL] cholesterol) at weeks 24 and 48; the incidence of hypoglycemic events and adverse events at weeks 12, 24, 36, and 48; satisfaction evaluation according to the Diabetes Treatment Satisfaction Questionnaire (DTSQ) [9, 10] at weeks 12, 24, 36, and 48; mean body weight, BMI, and body fat mass for 1 week prior to weeks 12, 24, 36, and 48; mean number of steps, burned calories, and walking distance for 1 month prior to weeks 12, 24, 36, and 48; and mean calorie intake for 3 consecutive days prior to weeks 12, 24, 36, and 48. For experimental group C, secondary outcomes also include CGM metrics, specifically the values of mean glucose; coefficient of variation (CV); and percentages of time in range (TIR), time above range (TAR), and time below range (TBR) for 1 week prior to weeks 12, 24, 36, and 48, and the number of educational interventions by medical staff at weeks 12, 24, 36, and 48.

### Sample size assumptions

Assuming a 0.3% difference in HbA1c values between the study groups (mean HbA1c 58 mmol/mol (7.44%) [standard deviation, 0.6%]) (effect size d = 0.50) according to previous studies, [7, 11] 78 subjects per group are required, with a two-sided alpha threshold of 0.05 and 80% power. Considering a dropout rate of 20%, the total number of patients required for this study is estimated to be 294 (98 patients per group) from the collective three hospitals.

### Data collection and intervention

#### Demographic and medical survey

The following demographic and medical information will be collected (Table 1): age, sex, smoking history, past and present medical history (e.g., duration of T2DM, hypertension, cardiovascular disease, liver disease, dyslipidemia), diabetes medication and other concomitant medication including steroids and statins during the 12 weeks prior to screening, and frequency of self-monitoring of blood glucose in the recent month.

**Table 1:** Study schedule

Study procedure	Screening	Baseline	12 weeks	24 weeks	36 weeks	48 weeks	Early termination
Visit	1	2	3	4	5	6	EDV
Visit window	−28 ~ 0 day	0	±14 days	±14 days	±14 days	±14 days	
Informed consent	**●**						
Demographic information and medical history	**●**						
Inclusion/exclusion criteria	**●**						
Physical examination	**●**						
Randomization		**●**					
Digital integrated healthcare platform application (Groups B, C)		**●**	**●**	**●**	**●**	**●**	**●**
Continuous glucose monitoring system application (Group C)		**●**	**●**	**●**	**●**	**●**	**●**
Continuous glucose monitoring system data collection (Group C)		**●**	**●**	**●**	**●**	**●**	**●**
Vital signs	**●**	**●**	**●**	**●**	**●**	**●**	**●**
Laboratory tests	**●**	**●**	**●**	**●**	**●**	**●**	**●**
Evaluation of hypoglycemia/severe hyperglycemia		**●**	**●**	**●**	**●**	**●**	
Satisfaction questionnaire (DTSQ)	**●**		**●**	**●**	**●**	**●**	**●**
Digital integrated healthcare platform data collection (Group B, C)		**●**	**●**	**●**	**●**	**●**	**●**
Monitoring and intervention (Group C)		**●**	**●**	**●**	**●**	**●**	**●**
Adverse event report		**●**	**●**	**●**	**●**	**●**	**●**
Concomitant drugs	**●**	**●**	**●**	**●**	**●**	**●**	**●**

### Anthropometric and vital sign measurements

At each clinical visit, vital signs including blood pressure, heart rate, and, body weight, and BMI will be collected.

### Laboratory tests

At baseline, week 24, and week 48, blood laboratory tests will be performed after 8 h of fasting. Fasting glucose, HbA1c, total cholesterol, HDL cholesterol, LDL cholesterol, triglycerides, and creatinine will be measured. At weeks 12 and 36, HbA1c and fasting glucose will be measured.

### Distribution and use of at-home measurement devices and the digital integrated healthcare platform

For subjects in experimental groups B and C, at-home measurement devices of a Bluetooth-enabled glucometer, scale with bioelectrical impedance analysis, sphygmomanometer, and watch-type pedometer will be provided. Subjects in group B will configure and apply these devices and the platform by themselves, while subjects in group C will be provided instruction regarding the configuration and use of the devices and the platform by medical staff during their hospital visit.

### Dietary data

For subjects in group B and group C, dietary information will be collected by the AI-based dietary management solution. From a photo of a meal taken prior to eating, the program recognizes the food items. When the subject enters the amount of each food they consume, the dietary information will be converted to food group and calories and recorded on the digital integrated healthcare platform.

### Data acquisition by the digital integrated healthcare platform

For subjects in group C, the following data will be collected from the indicated devices, all Bluetooth-enabled, and recorded on the digital integrated healthcare platform: glucose level from the glucometer; body weight, BMI, and body fat mass measured by a scale with bioelectrical impedance analysis; blood pressure by sphygmomanometer; number of steps, burned calories, and walking distance assessed by a watch-type pedometer; and intake calories assessed by the AI-based dietary management solution. Subjects in group C will be able to access their integrated data via the smartphone app, and medical staff can also access the data on the platform.

### Glucose data by continuous glucose monitoring system

Subjects in group C will apply a CGMS for 1 week prior to the hospital visit at weeks 12, 24, 36, and 48. CGM metrics comprise values of mean glucose; CV; and percentages of time in range (TIR), time above range (TAR), and time below range (TBR) during that week will be collected.

### Evaluation of hypoglycemia and adverse event assessment

At each clinical visit, incidence and frequency of hypoglycemia and severe hypoglycemic events in the previous 3 months will be assessed. Investigators must report all pregnancies or new severe adverse events.

### Questionnaire for satisfaction with diabetes treatment

All subjects will complete the DTSQ to assess the effect of the intervention at every visit.

#### Intervention

Subjects in group A will maintain routine diabetic care during the study period of 48 weeks. Subjects in group B will use a digital integrated healthcare platform by themselves to manage blood glucose during the study period, with no monitoring or intervention from medical staff.

Subjects in group C will be assisted by medical staff in installing the digital integrated healthcare platform when they visit their clinic and will apply the platform during the study period of 48 weeks. Investigators will continuously monitor all data collected from the digital integrated healthcare platform. Medical staff will motivate patients to maintain their self-care through ongoing personal monitoring and intervention. Participants of C group will be provided a copy of the food exchange table on the first day of the study. The daily calorie requirement for each participant is calculated based on the optimal body weight and normal activity level. Each subject in group C will receive a text message once a week via the digital healthcare platform. Clinical registered dietitians will send one of four types of nutritional intervention messages, which are categorized as warning, education, encouragement, or confirmation, based on blood glucose level, CGM metrics, body weight, blood pressure, activity level, and dietary records (Table 2). In addition, a nutritional education message will be sent every 4 weeks.

**Table 2:** Contents of interventional text messages

Message type	Examples
Warning	• Warning for excessive calorie intake, extra snacks, high amount of sugar or alcohol consumption. • Lack of daily activity • Imbalance of food intake based on food group: for example, heavy intake of fruit or grains and insufficient intake of protein.
Education	• Card-type mini-educational messages according to the weekly meal pattern; for example, food exchange table, nutrition facts, calories of restaurant food, GI index, and the relationship between exercise and level of blood glucose.
Confirmation	• Verification of previous warning and educational messages • Feedback on daily practice • Instruction and assessment of weekly practice content
Encouragement	• Encouraging message to minimize the drop-out rate • Encouraging message for positive changes in diet or body weight, etc.

A total of four 3-day intensive nutrition management periods are set at 12-week intervals during the 48-week intervention. Participants of C group are encouraged to complete 3-day dietary records and will receive a nutrition management text message every day based on the daily dietary records. The number of educational interventions provided by medical staff at weeks 12, 24, 36, and 48 will be collected.

### Data analysis

Baseline comparisons between groups for continuous variables will be performed using the two-sample t-test for normally distributed outcomes or Wilcoxon’s rank-sum test for non-normally distributed outcomes. Pearson’s chi-square or Fisher’s exact test will be used for categorical variables. Changes from baseline in each group will be compared by McNemar’s test for proportions and the paired t-test or Wilcoxon signed-rank test for means.

### Rescue therapy

In all three groups, clinicians can add or titrate diabetes medicine until week 24 if HbA1c ≥ 86 mmol/mol (10%) or symptoms of hyperglycemia or hypoglycemia develop. After week 24, diabetes medication can be changed if HbA1c ≥ 69 mmol/mol (8.5%) or symptoms of hyperglycemia or hypoglycemia develop. Additionally, in group C, clinicians may change the medication if frequent hypoglycemia (< 3.89 mmol/L (70 mg/dL), > 3% of total time of a day) or frequent hyperglycemia (> 9.99 mmol/L (180 mg/dL), > 30% of total time of a day) is reported on the CGMS.

### Discontinuation of subjects

Subjects may stop the intervention and be excluded if any of the following safety issues occur: 1) hyperosmolar hyperglycemic state (HHS) or 2) complicated systemic diseases that may have a significant impact on the clinical trial. Subjects also can be dropped if any of following situations occur: 1) the subject or legal representative requested to discontinue the study; 2) subjects who did not meet the selection criteria were included in the trial; 3) the subject does not or cannot comply with the visits and procedures specified in the trial plan; 4) follow-up loss of the subject; or 5) other problems that investigators consider to be inappropriate for continuing the trial.

## Discussion

In this study, the effect of a digital integrated healthcare platform will be investigated in patients with type 2 diabetes who have HbA1c 53–69 mmol/mol (7.0–8.5%) and body mass index (BMI) ≥ 23 mg/m^2^. We plan to apply a digital integrated healthcare platform using an artificial intelligence (AI)-based dietary management solution and a continuous glucose monitoring system (CGMS), and medical staff will continuously monitor the collected healthcare data and send regular educational messages via the platform to encourage efficient self-management of diabetes.

Previously, the efficacy of several smartphone apps for diabetes management was demonstrated via randomized controlled trial [5, 6]. However, even if the efficacy of apps has been demonstrated in clinical trials, the effect may decrease if the patient does not actively use the apps. In 2016, Payal Agarwal et al. [12] investigated the impact of “Bluestar,” an application developed for self-management of diabetes and approved by the Food and Drug Administration, among different clinical sites and health systems. They conducted a pragmatic, multicenter, randomized trial in three recruitment sites. They reported low usage of the app among participants, with a mean number of log-in days of 42.4 (SD 52.1) over 26 weeks, with 46.4% (51/110) of the participants using the app for 10 days or less, and the results showed no difference in HbA1c between the intervention and control groups [12].

One of the strengths of the present study is the use of an AI-driven dietary solution for the record of dietary intake. The image-assisted dietary assessment was shown in previous studies to reduce underreporting of dietary intake compared with traditional methods [13]. The dietary program in this study is expected to be more convenient and effective in that it recognizes all foods by a single capture utilizing the AI solution. Also, for user-friendliness, participants can see their various healthcare data, such as glucose level, body weight, blood pressure, daily activity, and dietary data, on the digital integrated healthcare platform app by connecting Bluetooth-enabled at-home measurement devices including a glucometer, scale, sphygmomanometer, and watch-type pedometer.

A randomized controlled study [14] that aimed to investigate the efficacy of a text message-based diabetes self-management support intervention (SMS4BG) in subjects with diabetes was conducted from 2015 to 2016 in New Zealand. The study showed a significantly greater reduction in HbA1c in the intervention group (mean − 8.85 mmol/mol) than in the control group (− 3.96 mmol/mol) (*p* = 0.007) after 9 months. The result suggests that text messages help diabetes patients with self-management. Furthermore, this current study, which utilizes the digital integrated healthcare platform, will involve health information monitoring and intervention via regular educational text messages from medical staff to provide feedback and encourage patients to continue self-management of diabetes. Thus, the contents of diabetes self-management will not be separated but linked to medical care in the clinic.

Therefore, the results of the current study of the digital integrated healthcare platform with an AI-driven dietary solution and CGMS can yield a positive impact on the self-management of diabetes in patients with type 2 diabetes mellitus.

## Data Availability

Not applicable.

## Abbreviations

AI: Artificial intelligence
Apps: Mobile (smartphone) applications
BMI: Body mass index
CGMS: Continuous glucose monitoring system
CV: Coefficient of variation
DM: Diabetes mellitus
DTSQ: Diabetes treatment satisfaction questionnaire
HDL: High-density lipoprotein
LDL: Low-density lipoprotein
TAR: Time above range
TBR: Time below range
TIR: Time in range
T2DM: Type 2 diabetes mellitus
